# Geriatrics Clinic Primary Care: Screening for Mental Health and Psychological Flexibility

**DOI:** 10.1093/geroni/igaa057.3142

**Published:** 2020-12-16

**Authors:** Monica Scicolone

**Affiliations:** The University of Alabama, Tuscaloosa, Alabama, United States

## Abstract

At baseline assessment, average level of symptoms for both depression (M = 3.75) and anxiety are low (M = 1.65). OLS regression analyses at baseline revealed psychological inflexibility (β = .474, p < .001) and anxiety symptoms (β = .334, p < .001) were significantly associated with depressive symptoms, and psychological inflexibility (β = .331, p = .002) and depression symptoms (β = .381, p < .001) were significantly associated with anxiety symptoms. These associations remained at year two in data collection. As of March 2019, there are 88 individuals with time two data for depressive symptoms (M = 2.99), and 79 individuals with time two data for anxiety symptoms (M = 1.34). Repeated measures ANOVA revealed that neither mean scores for depression nor anxiety changed significantly from time one to time two. As data collection continues into year three, multilevel modeling will be used to analyze longitudinal data. Part of a symposium sponsored by the Mental Health Practice and Aging Interest Group.

